# C-Terminal Binding Protein (CtBP) Activates the Expression of E-Box Clock Genes with CLOCK/CYCLE in *Drosophila*

**DOI:** 10.1371/journal.pone.0063113

**Published:** 2013-04-30

**Authors:** Taichi Q. Itoh, Akira Matsumoto, Teiichi Tanimura

**Affiliations:** 1 Graduate School of Systems Life Sciences, Kyushu University, Hakozaki, Fukuoka, Japan; 2 Department of Biology, Juntendo University School of Medicine, Inba-gun, Chiba, Japan; Ludwig-Maximilians-Universität München, Germany

## Abstract

In *Drosophila*, CLOCK/CYCLE heterodimer (CLK/CYC) is the primary activator of circadian clock genes that contain the E-box sequence in their promoter regions (hereafter referred to as “E-box clock genes”). Although extensive studies have investigated the feedback regulation of clock genes, little is known regarding other factors acting with CLK/CYC. Here we show that Drosophila C-terminal binding protein (dCtBP), a transcriptional co-factor, is involved in the regulation of the E-box clock genes. *In vivo* overexpression of dCtBP in clock cells lengthened or abolished circadian locomotor rhythm with up-regulation of a subset of the E-box clock genes, *period* (*per*), *vrille* (*vri*), and *PAR domain protein 1ε* (*Pdp1ε*). Co-expression of dCtBP with CLK *in vitro* also increased the promoter activity of *per*, *vri*, *Pdp1ε* and *cwo* depending on the amount of dCtBP expression, whereas no effect was observed without CLK. The activation of these clock genes *in vitro* was not observed when we used mutated dCtBP which carries amino acid substitutions in NAD^+^ domain. These results suggest that dCtBP generally acts as a putative co-activator of CLK/CYC through the E-box sequence.

## Introduction

Many organisms show circadian rhythms in physiology, metabolism, and behavior. These rhythms are controlled by an endogenous circadian clock [1]. In *Drosophila*, there are seven transcription factors among core components of the circadian clock. The transcriptional activator CLOCK/CYCLE heterodimer (CLK/CYC) binds to the E-box sequence in the promoter regions of clock genes *period* (*per*), *timeless* (*tim*), *vrille* (*vri*), *PAR domain protein 1ε* (*Pdp1ε*), and *clockwork orange* (*cwo*) to activate their transcription [1]–[3]. The product proteins of these genes feed back to control their own transcription. Three feedback loops are tightly interlocked to yield the circadian oscillation of clock genes’ products. In one loop, PER/TIM suppresses the function of CLK/CYC to generate the oscillation of their own transcription. In another loop, the transcription of *Clk* is mediated by VRI and PDP1ε which acts as a suppressor and an activator, respectively. In the other loop, CWO inhibits the transcription of clock genes to bind the E-box sequence. This interlocked feedback loops generate and maintain circadian rhythm in pacemaker cells in the *Drosophila* head and regulate circadian output pathways that control circadian rhythms in physiology, metabolism, and behavior. Although CLK/CYC is well known as the primary factor regulating the circadian oscillation of transcription of the core clock genes as well as output genes, little is known regarding other factors that act with CLK/CYC. Although NEJIRE (NEJ), a homolog of CBP/p300 [4], has been reported as a co-factor of CLK, conflicting reports have claimed that it acts as a co-activator [5] and co-repressor [6].

Drosophila C-terminal binding protein (dCtBP) [7], [8] is a homolog of human CtBP that binds to the C-terminal region of human adenovirus E1A proteins to negatively modulate an oncogenic transformation [9], [10]. dCtBP was initially reported as a transcriptional co-repressor functioning during embryonic development in *Drosophila* [11]. dCtBP forms complexes with Knirps, Snail and Hairy, all of which contain a DNA-binding domain, to suppress transcription of their target genes [11], [12]. The consensus sequences P-DLS-K in Knirps and Snail and PLSLV in Hairy have been identified as binding sequences of dCtBP [9], [11], [12]. Although dCtBP is well known to function as a repressor, a recent study reported that dCtBP may also function as an activator in the Wingless signaling pathway [13], [14]. In the adult brain, ubiquitous expression of dCtBP has been reported in virtually all neurons including pacemaker cells [15]. CtBP contains extensive homology with D-2-hydroxy acid dehydrogenases, including the conserved nicotinamide adenine dinucleotide domain (NAD^+^) and has dehydrogenase activity [16]. In mammal, NAD^+^ is associated with CLOCK/BMAL1 function through SIRT1 [17], and NAD^+^ and SIRT1 function as a molecular switch to modulate both expression of clock genes and metabolism [17]. We revealed that dCtBP acts as a putative co-activator of CLK/CYC in the transcription of a subset of the E-box clock genes both *in vivo* and *in vitro* and its NAD^+^ domain is essential for the activation.

## Results

### *dCtBP* Affects Circadian Locomotor Rhythm in *tim*-positive Cells

**Figure 1:**
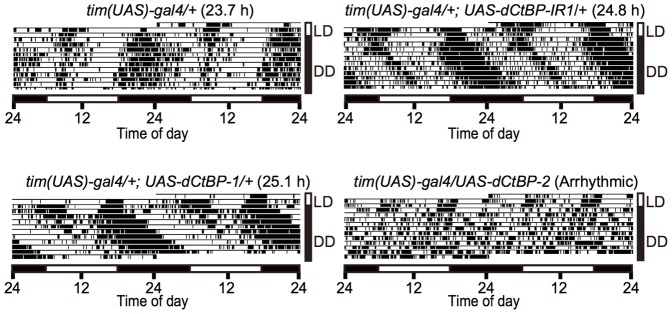
The actograms of *dCtBP*-knockdown and -overexpressing flies. Typical locomotor activity in the control (upper left), *dCtBP*-knockdown flies (upper right), and *dCtBP-*overexpressing flies (lower panels). The number in parentheses represents the free-running period of the corresponding flies. Adult flies were entrained to a 12-h light:12-h dark cycle (LD) for 3 days, and then kept in constant darkness (DD). Horizontal bars in white and black indicate times of light and dark, respectively, in LD. Vertical bar in white: LD; vertical bar in black: DD.

To screen new clock genes, we used the EP lines [18], which carries the *Upstream Activation Sequence* (*UAS*) insertion in the promoter region of a target gene. The EP lines were crossed with *tim(UAS)-Gal4* as a driver [19]. Because *tim* is expressed in virtually all clock-related cells [20], a target gene downstream of *UAS* can be activated by GAL4 in these tissues. This allowed us to screen for gene candidates which contribute to the circadian system, regardless of the tissue specificity of the target gene expression. We found *EP3352* strain carrying the *UAS* insertion in the promoter region of *dCtBP* altered circadian locomotor rhythm when it was crossed with *tim(UAS)-Gal4*. About 80% of *tim(UAS)-Gal4;EP3352* flies became arrhythmic; the remaining flies demonstrated lengthening of the circadian period to over 26 h (Table 1). Because homozygous *EP3352* flies were semi-lethal, the *UAS* insertion in the promoter region of *dCtBP* might affect the expression of the *dCtBP* gene. We newly established two lines of *UAS-dCtBP* transgenic flies, which enabled us to investigate the effect of *dCtBP* overexpression. *tim(UAS)-Gal4/+; UAS-dCtBP-1/+* flies demonstrated a period length of approximately 25.5 h, significantly longer than those of the corresponding parental strains (t test, *P<*0.05). The other overexpression flies, *tim(UAS)-Gal4/UAS-dCtBP-2*, became totally arrhythmic (Figure 1 and Table 1). To further check the effect of dCtBP overexpression in limited clock cells, we used *pdf-Gal4* in which GAL4 is expressed in a subset of pacemaker neurons [21]. Both *pdf-Gal4/Y;+;UAS-dCtBP-1/+* and *pdf-Gal4/Y;UAS-dCtBP-2/+* flies showed a significantly longer period than corresponding each parental strains (t test, *P<*0.05).

**Table 1:** Free-running periods of *dCtBP*-overexpressing and knockdown flies.

*lines*	Period(mean ± SEM)	N_R_	N_A_
*tim(UAS)-Gal4/+*	24.06±0.06	32	0
*pdf-Gal4/Y*	24.17±0.07	27	1
*EP3352/+*	24.00±0.08	8	0
*UAS-dCtBP-1/+*	23.95±0.04	21	2
*UAS-dCtBP-2*	23.88±0.06	30	0
*tim(UAS)-Gal4;EP3352*	26.30±0.38a,b	4	15
*tim(UAS)-Gal4/+;UAS-dCtBP-1/+*	25.51±0.24a,b	11	2
*tim(UAS)-Gal4/UAS-dCtBP-2*	–	0	43
*pdf-Gal4/Y;+;UAS-dCtBP-1/+*	25.23±0.21a,b	10	2
*pdf-Gal4/Y;UAS-dCtBP-IR2/+*	25.32±0.27a,b	12	4
*UAS-dCtBP-IR1/+*	24.11±0.06	29	0
*UAS-dCtBP-IR2/+*	24.02±0.06	34	2
*tim(UAS)-Gal4/+;UAS-dCtBP-IR1/+*	24.53±0.05a,b	63	6
*tim(UAS)-Gal4/+;UAS-dCtBP-IR2/+*	24.45±0.04a,b	58	1
*pdf-Gal4/Y;+;UAS-dCtBP-IR1/+*	24.46±0.11a,b	14	0
*pdf-Gal4/Y;UAS-dCtBP-IR2/+*	24.46±0.11a,b	40	1

N_R_: Number of rhythmic flies recorded.

N_A_: Number of arrhythmic flies recorded.

a significantly different from the period of the flies carrying the *tim(UAS)-Gal4* as a control (t test, *P*<0.05).

b significantly different from the period of the flies carrying the *UAS* sequence as a control (t test, *P*<0.05).

Neuron-specific knockdown of *dCtBP* also affected circadian locomotor rhythm, although the effect was relatively smaller than that of *dCtBP* overexpression (Table 1 and Figure 1). The periods of knockdown flies tested were slightly but significantly longer than the corresponding parental strains– (t test, *P<*0.05) regardless of the GAL4 driver. About 10% of flies demonstrated arrhythmicity in *tim(UAS)-Gal4/+;dCtBP-IR1/+* flies.

### Overexpression of *dCtBP* Increases the Expression Levels of a Subset of E-box Clock Genes

**Figure 2:**
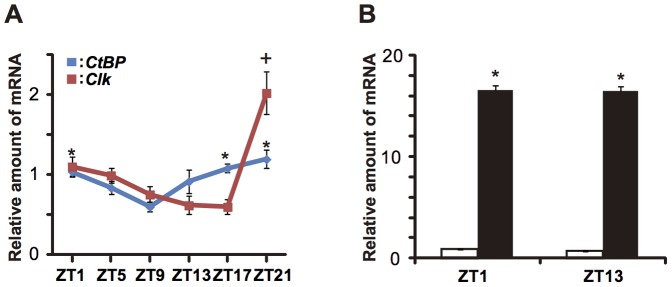
Temporal *dCtBP* expression in control and *dCtBP*-overexpressing flies. A: Temporal expression profile of *dCtBP* (blue) and *Clk* (red) in the head of adult control flies measured by quantitative PCR assay (Q-PCR). ZT1 and ZT13 correspond to 1 h from the onset of light-on and -off conditions in LD, respectively. *dCtBP* expression reveals a circadian rhythm peaking at the end of night phase. Cross indicates significant difference with trough level of *Clk* at ZT17 (Tukey’s test, *P*<0.05). Asterisks indicate a significant difference with the trough level of *dCtBP* at ZT9 (Tukey’s test, *P*<0.05). RNAs were sampled three times at each point, and error bars represent S.E.M. B: The expression level of *dCtBP* at ZT1 and ZT13 in control flies (white) and *dCtBP-*overexpressing flies (black). *dCtBP* expression was higher in *dCtBP*-overexpressing flies than control flies at each phase (*: t test, *P<*0.05). RNAs were sampled three times at each point, and error bars represent S.E.M. (n  = 3).

The daily expression profile of *dCtBP* in the fly head was measured by quantitative PCR analyses (Q-PCR). The expression level of *dCtBP* in the driver line as a control showed rhythmicity with a low amplitude (Figure 2A). The statistical analysis with Tukey’s test reveals that it peaks at the end of night phase, which is close to that of *Clk* [22], [23] (Figure 2A). The expression level of *dCtBP* was also determined at ZT1 and ZT13 in the *tim(UAS)-Gal4/UAS-dCtBP-2* flies, which showed arrhythmicity. The former corresponds to the trough phase of E-box clock genes expression, while the latter corresponds to the peak phase. The *dCtBP* expression level was 17-times higher than that of controls at both phases (Figure 2B).

**Figure 3:**
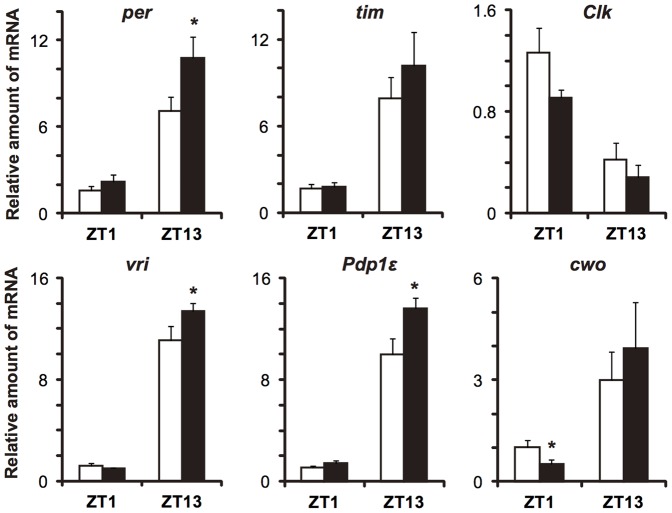
Expression levels of core clock genes in *dCtBP*-overexpressing flies. Relative mRNA levels of the indicated genes at the peak and trough phases were measured using a quantitative PCR assay (Q-PCR). Expression levels of *per, vri*, and *Pdp1ε* were higher in the *dCtBP* overexpression flies (black) than in control (white) at the peak phase. *dCtBP* overexpression decreased the expression levels of *cwo* at the trough phase. Asterisks indicate a significant difference from control values (t test, *P<*0.05). RNAs were sampled three times at each point, and error bars represent S.E.M.

Next, the expression levels of known clock genes were measured in this arrhythmic *dCtBP* overexpression flies at ZT1 and ZT13. In the case of *Clk*, whose expression is not controlled through an E-box [24], expression oscillated in antiphase to E-box clock genes. The levels of *per*, *vri*, and *Pdp1ε* increased at their peak phase, whereas that of *cwo* decreased at the trough phase (Figure 3). The expression level of *tim* showed no significant change at both phases (Figure 3).

**Figure 4:**
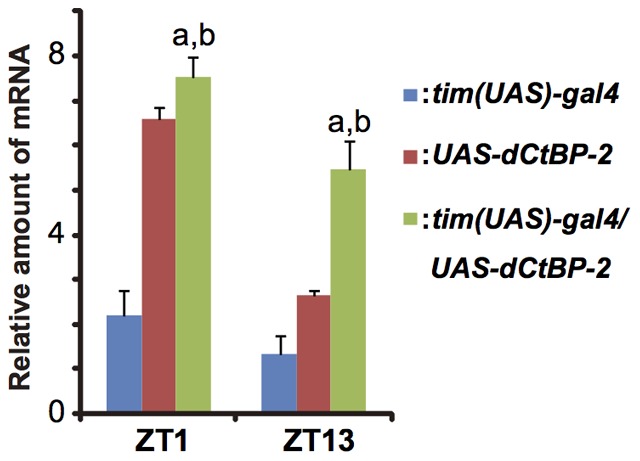
Expression level of an output gene, *takeout*, in *dCtBP*-overexpressing flies. Relative mRNA levels of *takeout* were measured at ZT1 and ZT13 using a quantitative PCR assay (Q-PCR). The blue, red and green bars represent the *tim(UAS)-Gal4*, *UAS-dCtBP-2* and *dCtBP* overexpression flies, respectively. The expression level in *dCtBP* overexpression flies was significantly different from that in *tim(UAS)-Gal4* (a: t test, *P<*0.05) and that of *UAS-dCtBP-2* (b: t test, *P<*0.05) at both phases. RNAs were sampled three times at each point and error bars represent S.E.M.

Then in order to investigate whether the effect of *dCtBP* overexpression can be observed in output genes, we quantified the expression level of *takeout* (*to*) [25] whose expression shows circadian rhythm [26], [27]. We compared the expression level of *to* in three groups of flies, *tim(UAS)-Gal4*, *UAS-dCtBP-2* and *tim(UAS)-Gal4/UAS-dCtBP-2*. *dCtBP* overexpression significantly increased *to* expression both at the peak and trough phases in *tim(UAS)-Gal4/UAS-dCtBP-2* flies as compared to those in the parental lines (t test, *P*<0.05). It seemed that the expression of *takeout* (*to*) maintain rhythmicity even in the arrhythmic flies (Figure 4).

These results suggest that *dCtBP* overexpression affects clock-related gene expression. In general, *dCtBP* overexpression activates the expression of E-box clock genes at the peak phase although there are some exceptions as we observed in *tim* and *cwo*. Interestingly, circadian expression rhythm seemed to persist in all clock-related genes we tested, although *dCtBP* overexpression flies became arrhythmic at the behavioral level.

### dCtBP Protein is a Putative Co-activator of CLK/CYC

**Figure 5:**
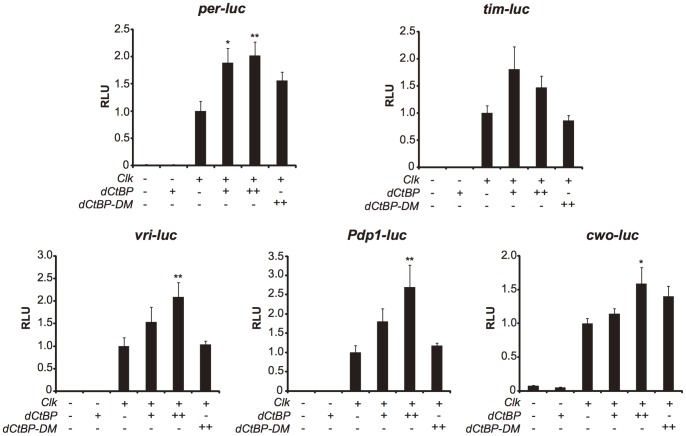
*dCtBP* regulates transcription of known clock genes with CLK/CYC. Relative luciferase activities of *per-luc*, *tim-luc*, *vri-luc, Pdp1-luc*, and *cwo-luc* in the presence of 0 (–) or 100 (+) ng *pAc5.1-dCtBP* alone, or 0 (–), 100 (+), 400 (++) ng *pAc5.1-dCtBP* (*dCtBP*), or 400 (++) ng *pAc5.1-dCtBP-G183A/G186A* (*dCtBP -DM*) in conjunction with 100 ng *pAct-Clk* are represented. The luciferase activity was normalized by the activity of *Renilla* luciferase as a control reporter, and then the activity was normalized by the activity of *pAct-Clk* alone. RLU means relative luminescence unit. *dCtBP* regulates the promoter activity of core clock genes. The difference between values without *Clk* was calculated by t test. The difference between the values with *Clk* was calculated by the Tukey’s test, and asterisks indicate significant differences between two values (*P<0.05 and **P<0.01). These experiments were performed independently three times (or four in some cases) and error bars represent S.E.M.

The *luciferase* assay in cultured *Drosophila* S2 cells was used to determine whether the gene-specific induction by dCtBP could be observed *in vitro*. First, we investigated whether dCtBP was able to regulate the E-box clock genes without CLK. S2 cells are reported not to express CLK [22]. Regulation by dCtBP was monitored by *promoter-luc*, in which firefly *luciferase* cDNA was linked to the promoter region including the E-box sequence of clock genes. None of those promoters were regulated by dCtBP without CLK (Figure 5). When we further co-transfected the plasmid to express CLK, *per-luc, vri-luc*, *Pdp1-luc* and *cwo-luc* were activated. This activation effect tended to correlate with the amount of dCtBP expression plasmid (Figure 5). However, we could not observe a significant increase of *tim-luc* under the expression of dCtBP with CLK (Figure 5). Thus, except for the case of *cwo-luc*, these results obtained *in vitro* are principally consistent with the results of dCtBP overexpression *in vivo*.

Next, in order to investigate whether these activations are regulated via the nicotinamide adenine dinucleotide domain (NAD^+^) -dependence of dCtBP, we supplied CLK with the mutated dCtBP which carries two amino acid substitutions in NAD^+^ binding region (dCtBP-G183A/G186A) [28]. The expression level of all E-box clock genes we tested with the mutated dCtBP was not significantly different from the value without an intact dCtBP.

## Discussion

We propose that dCtBP affects the expression of E-box clock genes. The most obvious evidence is that dCtBP acts as a co-activator of CLK as observed in *per*, *vri* and *Pdp1ε* expression *in vivo* and *in vitro*. The regulation mechanism is associated with CLK, because our results *in vitro* suggest that dCtBP have no effect without CLK (Figure 2A). dCtBP may bind to CLK/CYC through an unidentified domain because we could not find a consensus sequence [9], [11], [12] in CLK and CYC to bind with dCtBP (data not shown). Alternatively, more plausible possibility is that an unknown factor acts as a bridge between dCtBP and CLK/CYC. One candidate to act as such a mediator might be NEJIRE (NEJ), which has been reported to directly bind to CLK and function as its co-factor [5], [6]. In mammals CtBP is postulated to antagonaize CBP/p300 [29] which is a homolog of NEJ [4].

We found that the activation in E-box clock genes did not occur with the mutated dCtBP having amino acid substitutions in NAD^+^ binding domain [16], [28] (Figure 5). The mutated dCtBP might become unstable so that the protein no longer activates those genes [30]. Alternatively, our result suggests that this domain is important for the activation of those genes. In mammal, NAD^+^ is reported to modulate the rhythmic expression of clock genes downstream of CLOCK/BMAL1, which is a counterpart of CLK/CYC in *Drosophila*, through Sirt1 [17]. Although it is unknown whether NAD^+^ contributes to *Drosophila* circadian clock, the metabolic regulation of the circadian oscillator via the NAD-dependence is probably conserved between mammal and fly.

The expression patterns of all core clock genes seemed to maintain rhythmicity, even in *dCtBP* overexpressing flies that demonstrated arrhythmicity at the behavioral level (Figure 4). The up-regulation of core clock genes by *dCtBP* overexpression may induce arrhythmicity in the output pathway both at the molecular and behavioral levels. *Pdp1ε* is a leading candidate responsible for this loss of rhythmicity because it is known to function not only as a core clock gene but also as a regulator of output genes including *to* [25], [27], [31]. However, our results reveal that the expression levels of both *Pdp1ε* and *to* increased with remaining its rhythmicity even in behaviorally arrhythmic *dCtBP* overexpressing flies. Thus the responsible output genes that control locomotor rhythmicity may be more strongly affected by the increased level of *Pdp1ε* and lost rhythmicity. Alternatively, the dCtBP may directly regulate the expression of such output genes and arrhythmicity of dCtBP expression caused by overexpression induced arrhythmicity of expression in those genes.

Both overexpression and knockdown of *dCtBP* caused to lengthen circadian period. This is inconsistent with the general idea that an opposite effect on period could be induced by the excess and less product of the clock-related gene. Although we do not have a definitive explanation of this inconsistency at the present, it might be valuable to point out that recent reports reveal that dCtBP has dual roles as an activator and repressor of Wnt target genes [13], [14]. However, no reports to date have indicated an association between Wnt signaling and circadian gene expression in *Drosophila*. In addition, because the Wnt signaling pathway does not function in the S2 cells we used [32], we have not been able to obtain any supporting evidence at molecular level. The further extensive study is needed to determine whether dCtBP has dual roles as an activator and an repressor in *Drosophila* circadian clock. Given that CtBP in mammal is supposed to antagonize to CBP/p300, which is the counterpart of NEJ [29], our results may give a hint to dissolve the problem that there are conflicting reports that NEJ acts as a co-activator [5] and co-repressor [6] of CLK in *Drosophila*. Our study sheds new light on the regulation mechanism of the E-box clock genes by CLK/CYC and its co-factors.

## Experimental Procedures

### Fly Strains

*tim(UAS)-Gal4* strain [19] was used as the driver to knock down and overexpress *dCtBP*. *UAS-IR* lines [33] were established at the National Institute of Genetics. Knockdown flies were obtained by mating females of the driver line to males in each of the *UAS-IR* lines. The *EP3352* line [18] was obtained from the Harvard Stock Center. *UAS-dCtBP* transgenic lines were established by injection of *UAS-dCtBP* plasmid into *w^1118^* embryos (BestGene). *dCtBP*-overexpressing flies were obtained by mating the driver females to *EP3352* males or *UAS-dCtBP* transgenic males.

### Recording of Locomotor Activity Rhythm

Flies were kept on standard glucose-cornmeal medium under 12-h light:12-h dark cycles (LD) at 25°C. We measured the locomotor activity of the adult flies using *Drosophila* activity monitors (Trikinetics Inc.) for 3 days in LD cycles, then over 10 days in constant darkness (DD). A single fly was introduced into a measuring glass tube containing agar gel with 100 mg/ml glucose. The periods were calculated with a χ^2^ periodogram [34] programmed using the Matlab R2007b software (MathWorks Inc.).

### Q-PCR to Analyze Temporal Expression Levels of Clock Genes

The *tim(UAS)-Gal4* strain [19] was used as control. Control and *dCtBP*-overexpressing flies entrained for at least 3 days under LD were sampled three times at each point. Total RNA was isolated from 100 heads at each time point as described elsewhere [35]. cDNA was synthesized from 5 µg total RNA using Ready-To-Go T-Primed First-Strand Kit (Amersham) according to the standard protocol. Q-PCR was performed using Applied Biosystems 7300 and *Power* SYBR Green PCR Master Mix (Applied Biosystems). PCR reactions were performed with samples containing 1× *Power* SYBR Green PCR Master Mix (Applied Biosystems), 5 µM primers, and 1 µL synthesized cDNA in a 20 µL volume using the following amplification procedure: 10 min at 95°C, then 40 cycles of 15 s at 95°C, 30 s at 60°C, and 1 min at 72°C. *Gapdh2* expression levels were quantified and used as the internal control. We purified total RNA at each time point. Each RNA was used as a template to synthesize cDNA. We repeated these steps and obtained three different cDNAs at each point. One time-series of cDNAs were analyzed by Q-PCR at once with the primer sets in Table S1. The data finally obtained were calculated with the 2^−ΔΔCt^ Method [36] using the following equation, ΔΔCt = (Ct *target* – Ct *Gapdh2*) _ZT x_ – (Ct *target* – Ct *Gapdh2*)_ ZT1_. We confirmed that all primer sets we used didn’t yield any non-specific amplification by a melting curve analysis using the products of Q-PCR.

### Construction of Expression Plasmids

The coding sequence of *dCtBP* (see http://flybase.org/reports/FBgn0020496.html) was cloned into a *pAc5.1B-V5/His* plasmid (Invitrogen) by the SA-cloning method [37] using the sets of primers in Table S2.

To construct the *pAc5.1-dCtBP-G183A/G186A* plasmid, mutagenesis *of pAc5.1-dCtBP* was performed by site-directed mutagenesis PCR method using PCR with primers 5′- CTGGTGGGACTGGCCCGCATTGCTAGCGCCGTGGCCCTG-3′ and 5′- CAGGGCCACGGCGCTAGCAATGCGGGCCAGTCCCACCAG-3′.

To construct the *UAS-dCtBP* plasmid for transgenic flies, *dCtBP* was amplified by PCR using head cDNA in *w^1118^* as a template with primers 5′-AGCGAAATGGACAAAAATCTG-3′ and 5′-CTACGGCGCCTCCGTTGACT-3′ and cloned into *pCR2.1* vector (Invitrogen). To construct the *UAS-dCtBP* plasmid, the *dCtBP* PCR fragment in *pCR2.1* was doubly digested by *Spe*I and *Xba*I (New England Biolabs), purified, and cloned into the *Xba*I site of the *pUAST* plasmid [38].

### Luciferase Assay in *Drosophila* Cultured Cells

Cultured *Drosophila* S2 cells were plated in 24-well tissue culture plates with Shields and Sang M3 insect medium (Sigma-Aldrich) supplemented with 12.5% fetal bovine serum (Biowest) and antibiotics (12.5 U/mL penicillin, 12.5 mg/mL streptomycin; Invitrogen) and transfected by a standard method [22] using Effectene Transfection Reagent (QIAGEN) with 100 ng of each *promoter*-*luc* [39], [40] in the presence of 0 or 100 ng *pAc5.1- dCtBP* alone or 0, 100, or 400 ng *pAc5.1- dCtBP* in conjunction with 100 ng *pAct-Clk*. As a positive control for the luciferase assay, cells were transfected with 610 ng *pAc5.1B* empty vector (Invitrogen) with 10 ng *pAc5.1-Rluc*. Each *Luciferase* activity was measured 48 h after transfection as described elsewhere [39], [40]. The mean values were calculated from data obtained by three (or four in some cases) independent experiments.

## Supporting Information

Table S1 (DOC)

Table S2 (DOC)
