# Supplementary material for: C-Terminal Binding Protein (CtBP) Activates the Expression of E-Box Clock Genes with CLOCK/CYCLE in Drosophila
Source: PLoS One. 2013 Apr 30;8(4):e63113. doi: 10.1371/journal.pone.0063113 (PMC3640014; doi:10.1371/journal.pone.0063113)
Supplement: Table S1 — (DOC) [file pone.0063113.s001.doc]

Table S1. Primer sequences for quantitative PCR.

| Primer name | Sequence |
| --- | --- |
| *dCtBP-for.* | 5’-ACATACTGGCTAGCGAACATGGTG-3’ |
| *dCtBP-rev.* | 5’-GTGAGACGCAATCGGACTGGAAAA-3’ |
| *per-for.* | 5’-TACCCGCATCCTTCGCTTTTCT-3’ |
| *per-rev* | 5’-AATGCACCCGGCACCTTCT-3’ |
| *tim-for.* | 5’-ACTTTGCTGACAACTCCCACTTCC-3’ |
| *tim-rev.* | 5’-CTCCGCAGGGTCAGTTTAACGAA-3’ |
| *vri-for.* | 5’-AAGGGACTCCGGCATCTCCAT-3’ |
| *vri-rev.* | 5’-GCTTGCTTACTCCGCCAAGATCAT-3’ |
| *Clk-for.* | 5’-GTCAGTTCGCAAAGCCA-3’ |
| *Clk-rev.* | 5’-CGGCTCAAGAAATGTCG-3’ |
| *Pdp1-for.* | 5’-GCAACTGGTAATGGAAATGGTG-3’ |
| *Pdp1-rev.* | 5’-CTGTTCAAATGGTTGTGATGCTC-3’ |
| *cwo-for.* | 5’-ATCTGCGCCCAAGTGTACCT-3’ |
| *cwo-rev.* | 5’-TGCTTCTCCTCCATTTCCATTAAC-3’ |
| *takeout-for.* | 5’-GCCTTTTGGTCTCGGTGGAT-3’ |
| *takeout-rev* | 5’-GCCATCACCATACTTACAAGGTTTT-3’ |
| *Gapdh2-for* | 5’-CTACCTGTTCAAGTTCGATTCGAC-3’ |
| *Gapdh2-rev.* | 5’-AGTGGACTCCACGATGTATTCG-3’ |
