# Supplementary material for: C-Terminal Binding Protein (CtBP) Activates the Expression of E-Box Clock Genes with CLOCK/CYCLE in Drosophila
Source: PLoS One. 2013 Apr 30;8(4):e63113. doi: 10.1371/journal.pone.0063113 (PMC3640014; doi:10.1371/journal.pone.0063113)
Supplement: Table S2 — (DOC) [file pone.0063113.s002.doc]

Table S2. Primer sequences for construction of expression plasmid with SA-cloning.

| Primer name | Sequence |
| --- | --- |
| *dCtBP ATG-6* | 5’-AGCGAAATGGACAAAAATCTG-3’ |
| *dCtBP Stop* | 5’-CTACGGCGCCTCCGTTGACT-3’ |
| *dCtBP ATG-6 EFC1* | 5’-GGAGACATAGCGAAATGGACAAAAATCTG-3’ |
| *dCtBP Stop EFC2* | 5’-CGGGAAAGTCTACGGCGCCTCCGTTGA-3’ |
| *pAc5.1-forA* | 5’-CTGATGGAGCGGCTTTGTGTC-3’ |
| *pAc5.1-forA EFC1* | 5’-ATGTCTCCCTGATGGAGCGGCTTTGTGTC-3’ |
| *V5-S2* | 5’-ATGTCTCCCTGATGGAGCGGCTTTGTGTC-3’ |
| *V5-S2 EFC2* | 5’-ACTTTCCCGGAAGGTAAGCCTATCCCTAA-3’ |
